# Complete Genome Sequence of a Newcastle Disease Virus from a *Coturnix coturnix japonica* (Japanese Quail) Covey in India

**DOI:** 10.1128/genomeA.00374-14

**Published:** 2014-05-15

**Authors:** Srinivasan Bhuvaneswari, Krishnaswamy Gopalan Tirumurugaan, Jagaraj Cyril Jones, Kathaperumal Kumanan

**Affiliations:** a Department of Animal Biotechnology, ICAR-Niche Area of Excellence, Madras Veterinary College, Tamil Nadu Veterinary and Animal Sciences University, Chennai, Tamil Nadu, India; b Director of Research, Tamil Nadu Veterinary and Animal Sciences University, Madhavaram Milk Colony, Chennai, Tamil Nadu, India

## Abstract

The genome of a Newcastle disease virus isolated from a Japanese quail in 2003 is reported here. The genome is 15,192 nucleotides (nt) long, as found in the recent genotypes, and grouped as genotype VIIb, with a 6-nt insertion. This is the first report on the sequence of a genotype VII Newcastle disease virus (NDV) from India.

## GENOME ANNOUNCEMENT

Newcastle disease (ND) virus, a member of the *Paramyxoviridae* family and genus *Avulavirus*, has been shown to be present on six of the seven continents, and reports indicate the susceptibility of 241 different species, which includes 27 of the 50 orders of birds, to this virus. There has been a greater understanding of the diversity of ND virus (NDV) as a result of different vaccines that have been in use and also due to the availability of the whole-genome sequence information from different countries. An analysis of the genome had enabled the classification of the virus into 11 different genotypes (1), with recent reports of a novel genotype from South Africa (2). Preliminary analysis of the sequences available at GenBank for the Indian strain indicate the prevalence of genotypes II, IV, VI, and VII (3) and suggest that India is the only country to have genotype IV strains, which had been considered to have died out (4, 5).

Currently, whole-genome sequence information that has been generated by our laboratory is available on genotype II and IV field strains of NDV from India. We report here the whole-genome sequence information for an ND virus (ND-2K35) isolated in 2003 in 10-day-old specific-pathogen-free chicken eggs from a pooled homogenate of spleen and brain samples from a Japanese quail (*Coturnix coturnix japonica*) with clinical signs of diarrhea, lethargy, and nervous symptoms. This virus is a mesogenic strain, since it had an intracerebral pathogenicity index (ICPI) value of 1.98 and a mean death time of 67 h. Genotyping based on the 374-bp region of the fusion protein gene sequence confirmed it to belong to genotype VIIb. The whole-genome information was generated following assembly of the chromatograms (using the SeqMan module of the Lasergene version 7.1 [DNAStar, Inc., WI]) from three different clones using 36 different primer pairs, as reported in our earlier study (5), with the leader and trailer generated by rapid amplification of cDNA ends (RACE), as already described (6). The genotype of the virus was confirmed by generating the phylogenetic tree by employing MEGA 6.0 with maximum likelihood fits (using the Bayesian information criterion, maximum likelihood values, and Akaike information criterion corrected scores) (7).

The whole-genome sequence of ND-2K35/CHN/TN/2003 is 15,192 nucleotides, containing six genes in the order 3′-N-P-M-F-HN-L-5′, and has a six-nucleotide insertion (TCCCAC) downstream of the untranslated region of the nucleoprotein gene, as seen in the recent genotypes. The fusion protein cleavage site of this strain was ^117^SGGRRQKRF^119^, indicating it to be highly virulent, but the biological characteristics confirm the virus to be mesogenic.

All the residues that contribute to the sialic acid binding site and neutralizing epitopes as already reported were invariant in the Indian genotype VII strain. A comparison revealed isoleucine at position 69 of the fusion protein to be unique; however, this needs confirmation with more isolates (8). The predicted length of the hemagglutinin neuraminidase (HN) protein of NDV-2K35 is 571 amino acids, which is a common feature of most virulent NDV strains. Phylogenetic analysis of the 2K35/Chennai/Japanese quail whole-genome sequence also groups it in genotype VII and in the cluster of the subgroup genotype VIIb.

### Nucleotide sequence accession number.

The whole-genome sequence has been submitted to GenBank under the accession no. KF740478.

## References

[1] Maminiaina OF Gil P Briand FX Albina E Keita D Andriamanivo HR Chevalier V Lancelot R Martinez D Rakotondravao R Rajaonarison JJ Koko M Andriantsimahavandy AA Jestin V Servan de Almeida R 2010 Newcastle disease virus in Madagascar: identification of an original genotype possibly deriving from a died out ancestor of genotype IV. PLoS One 5:e13987. 10.1371/journal.pone.0013987 21085573 PMC2981552

[2] Susta L Jones ME Cattoli G Cardenas-Garcia S Miller PJ Brown CC Afonso CL 7 2 2014 Pathologic characterization of genotypes XIV and Newcastle disease viruses and efficacy of classical vaccination on specific pathogen-free birds. Vet. Pathol. 10.1177/0300985814521247 24510948

[3] Tirumurugaan KG Vinupriya MK Vijayarani K Kumanan K 2011 Analysis of the fusion protein cleavage site of Newcastle disease virus isolates from India reveals preliminary evidence for the existence of II, VI and VII genotypes. Indian J. Virol. 22:131–137. 10.1007/s13337-011-0044-1 23637515 PMC3550736

[4] Vijayarani K Muthusamy S Tirumurugaan KG Sakthivelan SM Kumanan K 2010 Pathotyping of a Newcastle disease virus isolated from peacock (*Pavo cristatus*). Trop. Anim. Health Prod. 42:415–419. 10.1007/s11250-009-9436-2 19763869

[5] Tirumurugaan KG Kapgate S Vinupriya MK Vijayarani K Kumanan K Elankumaran S 2011 Genotypic and pathotypic characterization of Newcastle disease viruses from India. PLoS One 6:e28414. 10.1371/journal.pone.0028414 22174801 PMC3235129

[6] Li Z Yu M Zhang H Wang HY Wang LF 2005 Improved rapid amplification of cDNA ends (RACE) for mapping both the 5′ and 3′ terminal sequences of paramyxovirus genomes. J. Virol. Methods 130:154–156. 10.1016/j.jviromet.2005.06.022 16076500

[7] Tamura K Peterson D Peterson N Stecher G Nei M Kumar S 2011 MEGA5: molecular evolutionary genetics analysis using maximum likelihood, evolutionary distance, and maximum parsimony methods. Mol. Biol. Evol. 28:2731–2739. 10.1093/molbev/msr121 21546353 PMC3203626

[8] Yu L Wang Z Jiang Y Chang L Kwang J 2001 Characterization of newly emerging Newcastle disease virus isolates from the People’s Republic of China and Taiwan. J. Clin. Microbiol. 39:3512–3519. 10.1128/JCM.39.10.3512-3519.2001 11574565 PMC88381

